# FAK Deletion Promotes p53-Mediated Induction of p21, DNA-Damage Responses and Radio-Resistance in Advanced Squamous Cancer Cells

**DOI:** 10.1371/journal.pone.0027806

**Published:** 2011-12-14

**Authors:** Kathryn Graham, Kim Moran-Jones, Owen J. Sansom, Valerie G. Brunton, Margaret C. Frame

**Affiliations:** 1 The Beatson Institute for Cancer Research, Garscube Estate, Bearsden, Glasgow, Scotland; 2 Edinburgh Cancer Research Centre, Institute of Genetics and Molecular Medicine, The University of Edinburgh, Edinburgh, Scotland; Faculdade de Medicina, Universidade de São Paulo, Brazil

## Abstract

Focal adhesion kinase (FAK) is a cytoplasmic tyrosine kinase that is elevated in a variety of human cancers. While FAK is implicated in many cellular processes that are perturbed in cancer, including proliferation, actin and adhesion dynamics, polarisation and invasion, there is only some limited information regarding the role of FAK in radiation survival. We have evaluated whether FAK is a general radio-sensitising target, as has been suggested by previous reports. We used a clean genetic system in which FAK was deleted from mouse squamous cell carcinoma (SCC) cells (FAK −/−), and reconstituted with exogenous FAK wild type (wt). Surprisingly, the absence of FAK was associated with increased radio-resistance in advanced SCC cells. FAK re-expression inhibited p53-mediated transcriptional up-regulation of p21, and a sub-set of other p53 target genes involved in DNA repair, after treatment with ionizing radiation. Moreover, p21 depletion promoted radio-sensitisation, implying that FAK-mediated inhibition of p21 induction is responsible for the relative radio-sensitivity of FAK-proficient SCC cells. Our work adds to a growing body of evidence that there is a close functional relationship between integrin/FAK signalling and the p53/p21 pathway, but demonstrates that FAK's role in survival after stress is context-dependent, at least in cancer cells. We suggest that there should be caution when considering inhibiting FAK in combination with radiation, as this may not always be clinically advantageous.

## Introduction

Radiotherapy is a mainstay of cancer therapy in multiple disease contexts, but treatment is not always curative. A great deal of effort is directed not only at improving the delivery of radiotherapy by increasingly sophisticated spatial and dosimetric methods, and also to identify combination strategies to improve radiation responses. In regard of the latter, ionizing radiation can promote activation of receptor and non-receptor tyrosine kinases (TKs), and modulation of cytoprotective influences, such as increased DNA repair, proliferation and reduced apoptosis [1], [2], [3], [4], [5], [6], [7]. Since these responses contribute to cellular radio-resistance, which can obviously limit the effectiveness of radiotherapy in cancer treatment, understanding the contribution of TKs may provide new molecular targets for radio-sensitisation, and potentially improve tumour responses. One example is the Epidermal Growth Factor Receptor (EGFR), which is the current most extensively studied TK in this context. Strong preclinical evidence implies a capacity of EGFR inhibition to enhance the anti-tumour effects of ionizing radiation, and this has translated into the clinical setting based on results of a Phase III trial in head and neck cancer [8], [9]. This demonstrates the importance of robust intervention strategies to establish whether particular TKs contribute to cellular radio-sensitivity, or to radio-resistance.

In contrast to the emerging evidence for EGFR, the role of other TKs, especially non-receptor TKs, is less clear. Focal Adhesion Kinase (FAK) is located at sites of integrin adhesion from where it transduces signals into cells that control multiple cancer-associated properties, including adhesion and actin dynamics, migration, invasion, angiogenesis, protection of cells from suspension-induced cell death (sometimes termed anoikis) and proliferation in 3-dimensions [10], [11], [12], [13], [14], [15], [16], [17]. FAK is often over-expressed in human cancer [18], [19], [20], [21], and plays a role in tumorigenesis, as demonstrated in multiple tissue types *in vivo* [22], [23], [24], [25], [26], [27], [28]. We previously showed that FAK deletion inhibits mouse skin cancer development and malignant progression, and that FAK deletion promotes apoptotic death of normal skin keratinocytes in culture [25]. More recently, we have also made use of the K14-Cre-ER^T2^/f*lox*-FAK mouse system to derive squamous cancer cells (SCC) from chemically-induced tumours [29], [30]. FAK deletion causes multiple defects, including impaired polarization and responses to directional cues, such as chemotactic invasion, as well as impaired growth in 3-dimensions (although growth on 2-D plastic is unaffected) and delayed growth as xenografts *in vivo* [29], [30].

FAK mediated pro-survival functions are thought to play an important role in cancer cell survival, and that this likely involves the p53 pathway [31]. Moreover, the FAK promoter contains p53 responsive elements and can be down-regulated by DNA-damage in a p53-dependent manner, while FAK expression correlates with mutant p53 in breast cancer [32], [33], [34]. There is also *in vitro* and *in vivo* evidence demonstrating that FAK knock-down can sensitise cells to cytotoxic chemotherapy [2], [35], [36], [37], [38], [39], [40], [41]. In contrast, there are relatively few studies on the role of FAK in radiation sensitivity. FAK phosphorylation is induced following exposure to ionizing radiation *in vitro* [42], although this may only have been a transient stress response as FAK's role was not explore. However, there is one report that siRNA-mediated FAK knock-down promotes radio-sensitisation in pancreatic cancer cells [43], although the underlying mechanism is unclear. Additionally, over-expression of FAK in HL-60 cells confers marked resistance to a variety of apoptotic stimuli, including ionizing radiation [44], all suggesting that inhibition of signaling through FAK is likely to promote radio-sensitivity. Here we have used a clean genetic deletion/reconstitution system to test FAK's role in cellular radiation response *in vitro* and *in vivo*, specifically in FAK-deficient SCC cells (and their FAK-expressing counterparts), and begin to dissect out the underlying mechanism.

## Results

**Figure 1:**
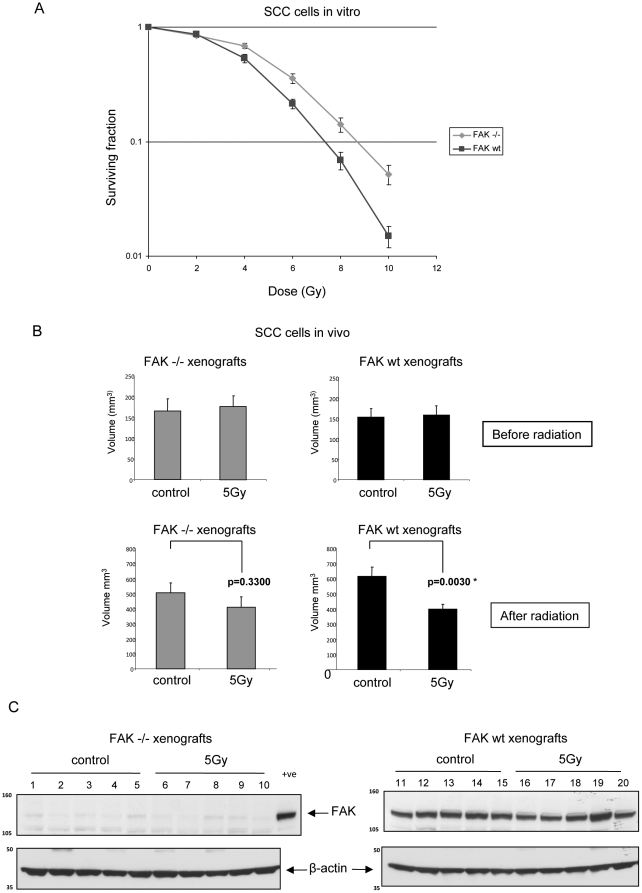
FAK deficiency is associated with increased radioresistance in SCC cells. (**A**) Subconfluent populations of FAK −/− and FAK wt cells were trypsinised and diluted in growth medium to a final concentration that would permit single colony growth. 100 µl of this suspension was added to each well of a 96 well plate. Following incubation for 6 hours to allow cell attachment the plates were irradiated with 0, 2, 4, 6, 8 or 10 Gy. The cells were analysed in triplicate for each radiation dose. After 7 days, the number of colonies per plate was counted and the surviving fraction calculated. The graphical representation shown represents the mean ± SEM from three separate experiments. Surviving fractions at each dose of radiation were compared with un-irradiated cells by student's unpaired t-test, n = 9. (**B**) 2×10^5^ FAK −/− and FAK wt cells were injected subcutaneously into the right flank of female nude mice. Xenografts were allowed to reach approximately 150 mm^3^. The animals were then irradiated with 5 Gy whole body irradiation or mock irradiated. After 7 days the xenografts were measured and the mice were sacrificed. The mean xenograft volumes ± SEM before radiation (upper panels) and after radiation (lower panels) are shown. Statistical analysis of mock irradiated versus irradiated volumes at 7 days was assessed by student's unpaired t-test, * denotes p<0.05, n = 10. (**C**) Protein extracts were prepared from the xenografts, separated by SDS-PAGE, transferred to nitrocellulose, and blotted with anti-FAK (upper) and anti-β-actin (lower) antibodies. A sample of five distinct extracts from each group is shown. A positive control (FAK wt cell extract) was added to the final lane of the FAK −/− xenograft samples.

**Figure 2:**
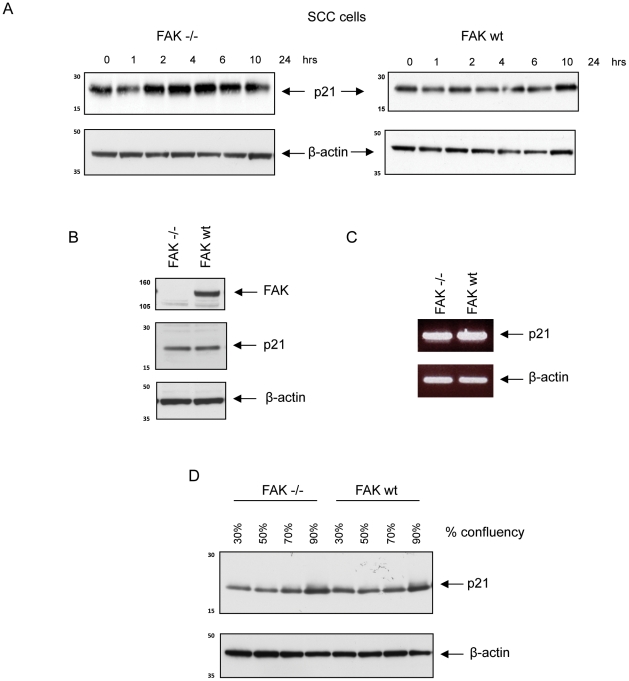
Ionising radiation results in p21 induction in FAK −/− cells but not in FAK wt cells. (**A**) FAK −/− and FAK wt cells were irradiated with 5 Gy at 70% confluence and lysates prepared at the indicated time points. Immunoblotting was then performed with anti-p21 (upper panel), and anti-β-actin (lower panel). (**B**) Protein extracts were prepared from subconfluent FAK −/− and FAK wt cell populations, separated by SDS-PAGE, transferred to nitrocellulose, and blotted with anti-FAK (upper panel), anti-p21 (middle panel) and anti-β-actin (lower panel) antibodies. (**C**) RNA was extracted from subconfluent FAK −/− and FAK wt cells, PCR performed and product analysed. β-actin loading is also shown (lower panel). (**D**) Protein extracts were prepared from FAK −/− and FAK wt cell populations at the level of confluency indicated. Immunoblotting was then performed with anti-p21 (upper panel) and anti-β-actin (lower panel) antibodies.

We derived SCC cells from chemically-induced squamous cell cancers in mice that expressed a *floxed* form of the ATP-binding coding exon of *fak* under the control of skin-specific (K14) *Cre* recombinase fused to the estrogen-receptor [25]. Excision of *floxed*-*fak* upon a single treatment with 4-hydroxy-tamoxifen (4-OHT) resulted in complete FAK protein deficiency [29], [30] (see also **Fig. 1C** and **2B**), which we could reverse by re-expressing wt FAK, allowing us to study how cancer cells cope with severe perturbation of the integrin/FAK signalling pathway. To assess radio-sensitivity, a limiting dilution clonogenic assay was performed comparing FAK −/− with FAK wt cells at increasing doses of radiation up to 10 Gy. This revealed that the complete absence of FAK in these cells was associated with increased radio-resistance *in vitro* (**Fig. 1A**). A statistically significant difference in surviving fraction was seen at doses of 4 Gy, 6 Gy, 8 Gy and 10 Gy (p values of 0.0136, 0.0097, 0.0045, and 0.0036 respectively, analysed by student's unpaired t-test, n = 9).

We also tested whether FAK influenced radio-sensitivity *in vivo*, by comparing FAK −/− and FAK wt SCC xenografts. 2×10^5^ cells were injected subcutaneously into the right flank of female nude mice and the animals were either irradiated with 5 Gy (in the form of whole body irradiation) or mock irradiated when the xenografts reached approximately 150 mm^3^. This size was selected as the FAK −/− tumours had overcome an initial delay in their growth *in vivo*, and their proliferation rate at this point did not significantly differ from their FAK wt counterparts. Previous studies demonstrated that the CD1 strain of nude mice could tolerate 5 Gy total body dose for 10–14 days. After 7 days, the xenografts were measured and the animals were sacrificed. Tumour volumes were calculated before and after 5 Gy irradiation or mock irradiation, and analysed by student's unpaired t-test. A statistically significant reduction in tumour volume was observed in the irradiated FAK wt xenografts compared with the mock-irradiated controls (p = 0.0030, n = 10), but this was not replicated in the FAK −/− xenografts (p = 0.3300, n = 10) (**Fig. 1B**). Protein extracts were prepared from 5 mice in each group and subjected to western blotting to confirm the level of FAK expression in FAK −/− and FAK wt tumours (**Fig. 1C**). The low levels of FAK present from FAK −/− tumour-derived material is likely from the small amount of stromal or immune infiltrate (**Fig. 1C**).

The SCC cells we used here expressed wild type p53 (confirmed by sequencing (not shown)), and we identified a FAK-dependent difference in induction of the p53 target gene, p21, after irradiation (**Fig. 2**; see also later). Specifically, induction of p21 was evident by 2 hours after treating FAK −/− cells with 5 Gy irradiation; by contrast, p21 was not induced when FAK was present (**Fig. 2A**). Interestingly, basal levels of p21 protein and mRNA in sub-confluent populations of both FAK −/− and FAK wt cells were similar (**Fig. 2B and 2C**, respectively), while p21 levels were elevated in both cell lines with increasing confluency (**Fig. 2D**). This was in keeping with the widely accepted role for p21 in contact-induced cell cycle arrest (**Fig. 2D**), and demonstrated that a different stimulus was able to increase p21 levels irrespective of FAK status. To ensure the discrepancy in induction of p21 following exposure to ionizing radiation was not related to differences in cell density, care was taken in all experiments to ensure that cells were irradiated at comparable confluency, typically 70%.

**Figure 3:**
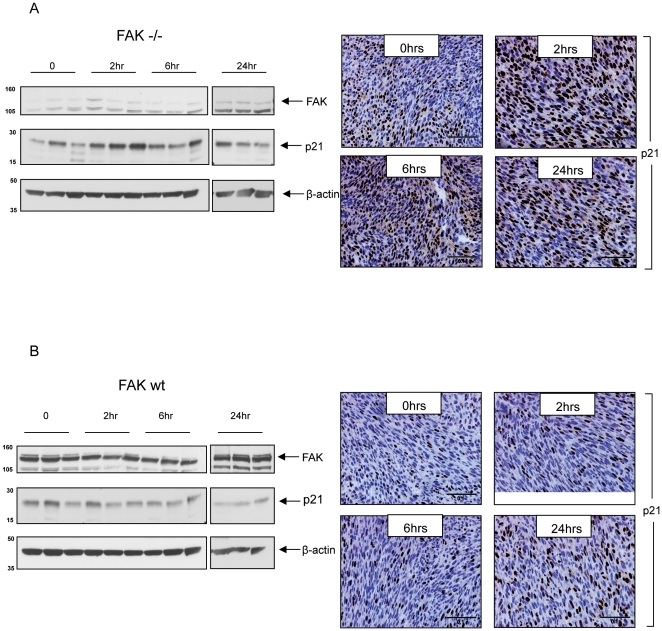
Ionizing radiation results in p21 induction in FAK deficient xenografts. (**A**) FAK −/− SCC cells or (**B**) FAK wt SCC cells, were injected subcutaneously into the right flank of female nude mice. When xenografts reached approximately 500 mm^3^, mice were irradiated with 5 Gy and sacrificed at 0, 2 hrs, 6 hrs, and 24 hrs (n = 3 per group). Half of the xenograft was fixed in formalin then embedded in paraffin and the other half was snap frozen in liquid nitrogen. Protein extracts were prepared from the frozen sections, separated by SDS-PAGE, transferred to nitrocellulose, and blotted with anti-FAK (upper blot panel), anti-p21 (middle blot panel), and anti-β-actin (lower blot panel). The paraffin embedded sections were stained with p21 and the p21-positive cells visualised by IHC (right panels). Representative bright field images of p21 stained tissue at 0, 2 hrs, 6 hrs, and 24 hrs post radiation are shown (scale bar, 0.1 mm).

The FAK-dependence of p21 regulation was reproduced *in vivo*. Specifically, nude mice were injected subcutaneously with 2.5×10^5^ FAK −/− or FAK wt cells, xenografts were allowed to establish, and the animals were then irradiated with 5 Gy irradiation when tumours reached approximately 500 mm^3^ in volume. Mice were sacrificed at 0, 2 hrs, 6 hrs, and 24 hrs after irradiation (n = 3 per group) and p21 levels were assessed by both western blotting of tumour lysates and immunohistochemistry (IHC) staining of paraffin embedded tissue. The FAK −/− xenografts exhibited an increase in p21 protein levels as early as 2 hours post irradiation (**Fig. 3A**, left panels); p21 levels appeared maximal around this time. The increase in p21 was also visible by IHC (**Fig. 3A**, right panels). Mean p21 positivity (based on scoring of 20 fields) was analysed across all time points and this demonstrated a significant difference in the p21 levels in the tumours of irradiated *versus* un-irradiated animals (Kruskal-Wallis, p = 0.038, n = 3). Further, individual comparison of the separate time points illustrated a statistically significant increase in p21 scoring compared with baseline levels (Mann Whitney, p = 0.0404, n = 3). In contrast, the FAK wt xenografts did not demonstrate any consistent increase in p21 levels at any of the time points examined. Western blotting of FAK wt-expressing tumour lysates confirmed the presence of FAK, but there was no appreciable increase in p21 protein levels (**Fig. 3B**). Further analysis of IHC (**Fig. 3B**, right panels) confirmed there was no significant increase in p21 expression at 2 hrs (p = 0.6625), 6 hrs (p = 0.6625), or 24 hrs (p = 0.3827) (Mann Whitney, n = 3), when compared with controls.

**Figure 4:**
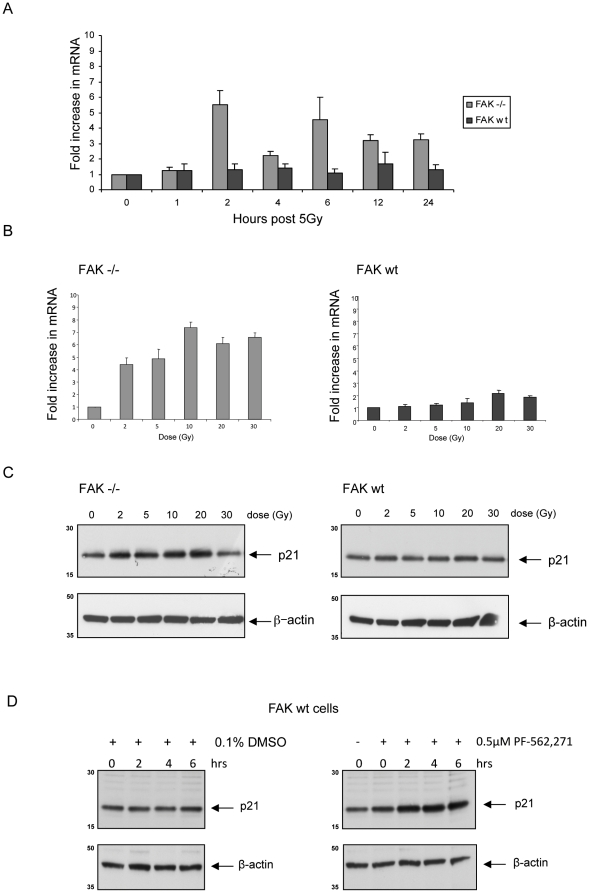
p21 induction following radiation is regulated at transcriptional level in FAK −/− cells. (**A**) RNA was extracted from subconfluent FAK −/− and FAK wt cell populations at various time points after 5 Gy irradiation. qRT-PCR analysis was then performed in triplicate. Fold increase in p21 mRNA levels was calculated using the ddC(t) method with β-actin as a loading control. Graphical representation of combined mean ± SEM from three experiments is demonstrated. (**B**) RNA was extracted from sub-confluent FAK −/− and FAK wt cell populations 2 hours after 0, 2, 5, 10, 20, and 30 Gy irradiation. cDNA was then generated and qRT-PCR for p21 performed as outlined above. (**C**) Protein extracts were prepared from FAK −/− and FAK wt cell populations 2.5 hours after exposure to various doses of radiation. The extracts were separated by SDS-PAGE, transferred to nitrocellulose, and blotted with anti-p21 (upper panels) and anti-β-actin (lower panels). (**D**) FAK wt cells were incubated for 2 hours with either the FAK inhibitor PF-562,271 at 0.5 µM, or 0.1% DMSO only, then irradiated with 5 Gy. Protein extracts were prepared at 0, 2, 4, and 6 hours and immunoblots probed with anti-p21 (upper panels), and anti-β-actin (lower panels). Representative immunoblots from 0.1% DMSO (left) and 0.5 µM drug treated (right) cell populations are shown.

We extracted RNA from sub-confluent populations of FAK −/− and FAK wt cells at various time points following 5 Gy irradiation, and qRT-PCR was performed using primers for endogenous p21. There was a biphasic increase in p21 mRNA levels, peaking at 2 hours and 6 hours post irradiation in FAK −/− cells; by contrast p21 mRNA levels were not induced in the FAK wt cell line after irradiation (**Fig. 4A**). RNA was also extracted from both cell lines 2 hours after a range of radiation doses (0, 2, 5, 10, 20, and 30 Gy) and analysed by qRT-PCR. We found that p21 mRNA levels increased in FAK −/− SCC cells in a dose-dependent fashion (range from 2 Gy to 10 Gy); further dose escalation did not result in further increased p21 mRNA. The dose-dependent increase in p21 transcription was attenuated upon re-expression of FAK wt in the FAK-deficient SCC cells (**Fig. 4B**). In parallel experiments, we found that increased steady state levels of p21 protein were evident after irradiation at various doses in FAK −/− SCC cells, and that this was attenuated when FAK expression was restored (**Fig. 4C**, compare left and right panels). To complement the genetic deletion of FAK, we also used a FAK kinase inhibitor (PF-562,271; [45]) at a dose of 0.5 µM (which is optimal for inhibition of FAK kinase activity in these cells (not shown)) for 2 hours prior to irradiation, and collected protein lysates for immunoblotting at 0, 2, 4, and 6 hours after 5 Gy. p21 levels were visibly increased by 2 hours after radiation in 0.5 µM PF-562,271 treated FAK wt cells when compared to untreated cells (**Fig. 4D**).

**Figure 5:**
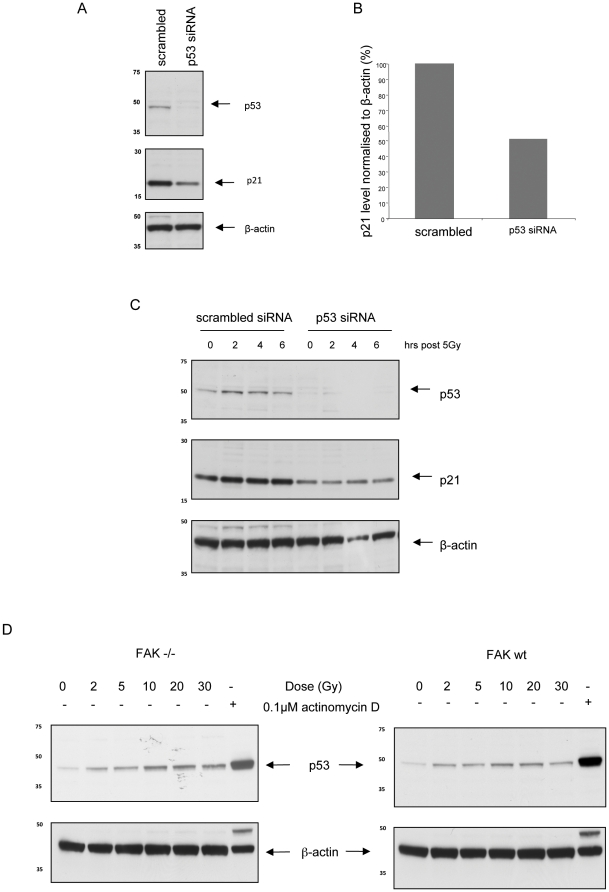
p21 induction in FAK −/− cells is p53 dependent. (**A**) FAK −/− cells were transfected with 100 nM siRNA (either scrambled pool or p53 siRNA) at 50% confluency. After 24 hours, protein extracts were prepared and immunoblots probed with anti-p53 (upper panel), anti-p21 (middle panel) and anti-β-actin (lower panel). (**B**) Densitometry comparing p21 levels in FAK −/− protein extracts treated with either a scrambled pool of siRNA or p53 siRNA was performed. The p21 protein levels were normalised to β-actin and results shown are representative of one of three separate experiments. (**C**) FAK −/− cells at 50% confluency were transfected with either 100 nM scrambled siRNA or 100 nM p53 siRNA, incubated for 24 hours, then irradiated with 5 Gy. Lysates were collected at the time points indicated and immunoblots probed with anti-p53 (upper panel), anti-p21 (middle panel) and anti-β-actin (lower panel). (**D**) Protein extracts were prepared from FAK −/− and FAK wt cells 2 hours after exposure to various doses of radiation (0–30 Gy). The extracts were separated by SDS-PAGE and immunoblots probed with anti-p53 (upper panels) and anti-β-actin (lower panels). The right lane in each gel contains protein extracts from FAK −/− or FAK wt cells exposed to overnight treatment with 0.1 µM of actinomycin D.

As expected, p21 steady state levels in the SCC cells were at least partly dependent on p53, and radiation-induced p21 in SCC cells was inhibited by knock-down of p53 using siRNA (**Fig. 5A–C**). However, we also noted that p53 induction after irradiation was not particularly strong and was similar in both FAK −/− and FAK wt SCC cells (**Fig. 5D**), indicating that the presence of FAK was leading to some uncoupling of p53 and p21 induction, and sensitivity to irradiation in these cancer cells. Densitometry was carried out to quantify fold changes in p21 and p53 protein levels after irradiation of FAK-proficient and FAK-deficient SCC cells (**Figure S1**). These findings imply that substantially increased transcription and expression of p21 protein occurs in FAK −/− cells after clinically relevant doses of ionizing radiation, and that this response is blunted by the presence of FAK. Thus, FAK functions in these advanced cancer cells to suppress the p53-dependent transcription of p21 after irradiation. This is not visibly linked to differential induction of cell cycle arrest (Figure S3 (determined as described in Methods S1)) or apoptosis, which is difficult to detect after SCC cell irradiation as judged by lack of sub-G1 DNA content (not shown). This is despite differential regulation of expression of the p53 target gene PUMA (Figure S4) that can be associated with apoptosis.

Previous work has established clear links between FAK and p53 that promotes survival after stress-induced signalling (in cells that lack p21), *via* the FAK FERM domain binding to p53 in the nucleus, facilitating p53 degradation and survival [46]. Therefore, we immunoprecipitated FAK from lysates of FAK wt SCC cells before and after 5 Gy irradiation, and immunoblotted for p53 and for Src (as a positive control). As expected, Src was bound to FAK, and this was unaltered by irradiation (**Figure S2A** (determined as described in Methods S1)). However, we found that FAK did not interact with p53 (**Fig. S2A**). Lysates were probed for FAK, Src and p53 to ensure equal loading (**Fig. S2B**). We also found that p53 was efficiently translocated to the nucleus in both FAK −/− and FAK wt cells after irradiation (not shown).

**Figure 6:**
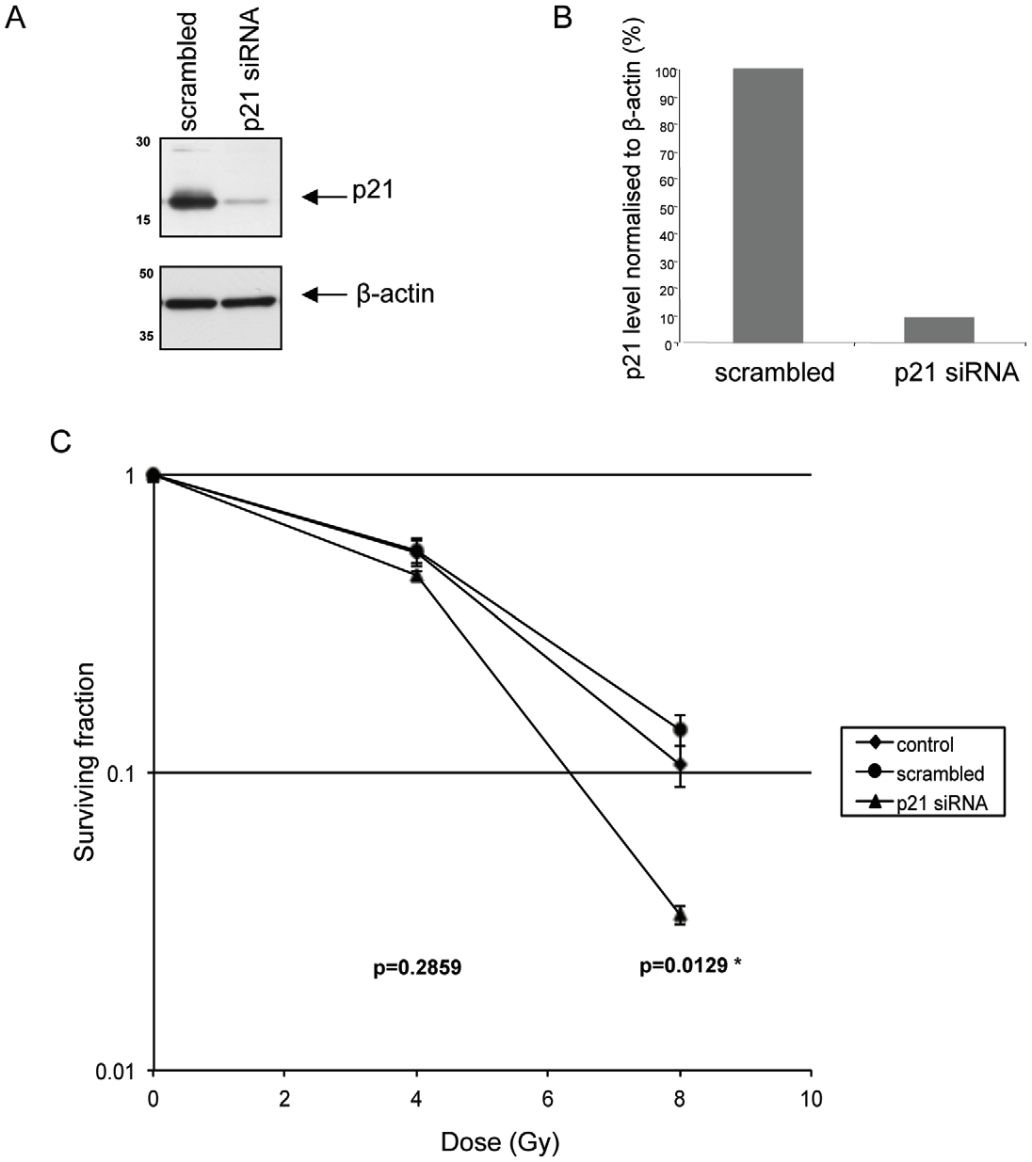
p21 knockdown increases radiosensitivity in FAK −/− cells. (**A**) FAK −/− SCC cells were transfected with 100 nM siRNA (either a scrambled pool or p21 siRNA) at 50% confluency. Following incubation for 24 hours, protein extracts were immunoblotted and probed with anti-p21 (upper panel) and anti-β-actin (lower panel). (**B**) Densitometry comparing p21 levels in FAK −/− protein extracts treated with either a scrambled pool of siRNA or p21 siRNA was performed. The p21 protein levels were normalised to β-actin level and results shown are representative of one of three separate experiments. (**C**) FAK −/− cells were mock transfected or transfected with 100 nM of either a scrambled siRNA pool or p21 siRNA at 50% confluency. After 24 hours the cell populations were trypsinised and diluted in growth medium to a final concentration that would permit single colony growth. 100 µl of this suspension was then added to each well of a 96 well plate. Following incubation for 6 hours to allow cell attachment the plates were irradiated with 0, 4, or 8 Gy. The plates were set up in triplicate for each radiation dose. After 7 days the number of colonies per plate was counted and the surviving fraction calculated. The graph shows the mean ± SEM from three separate experiments. Surviving fractions of p21 siRNA treated cells were compared with scrambled siRNA treated cells at each dose of radiation and statistical significance assessed by student's unpaired t-test, * denotes p<0.05, n = 9.

Since p21 has been associated with both resistance and sensitivity to DNA damaging agents, including ionizing radiation, we next depleted p21 using siRNA. We achieved around 90% knock-down of p21 in SCC cells (**Fig. 6A and 6B**). Clonogenicity was then assessed at 0, 4, and 8 Gy and comparison made between cell populations transfected with p21 siRNA, scrambled siRNA or control cell populations which had been mock transfected. We found a significant difference in surviving fraction at 8 Gy (p = 0.0129) between the scrambled siRNA- and p21 siRNA-treated cells, such that p21 promoted radio-resistance in the SCC cells (**Fig. 5C**). There is therefore a strong link between radiation-induced p21 in FAK-deficient cells (but not in their FAK-expressing counterparts) and the finding that FAK loss induces radio-resistance in which p21 has a causal role in the SCC cells.

**Figure 7:**
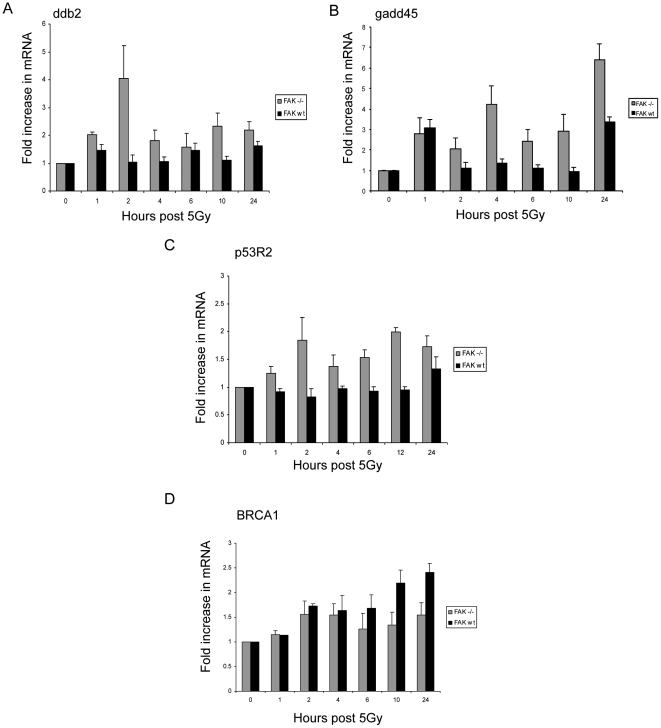
Induction of some p53 target genes involved in DNA repair is modulated by FAK. RNA was extracted from subconfluent FAK −/− and FAK wt cell populations at various time points after 5 Gy irradiation. qRT-PCR analysis was performed using primers directed against Ddb2 (**A**), gadd45 (**B**), p53R2 (**C**) and BRCA1 (**D**), and these were all normalised to β-actin.

Finally, we assessed whether FAK deficiency affected more general features associated with DNA damage. We found that mRNA levels of a number of p53 target genes, which are involved in repair following ionizing radiation, namely *gadd45*, *p53R2* and *ddb2*, and are known to be down-regulated by c-Myc [47], were stimulated in FAK −/− SCC cells, but consistently less so in their FAK-expressing counterparts (**Fig. 7**). This was particularly true for *ddb2* at the 2 hour time point, for *gadd45* at later time points, and for *p53R2* throughout the 0–24 hours after irradiation (**Fig. 7A, B and C**). Since we showed that FAK is required for c-Myc up-regulation downstream of *Apc* deletion in mouse intestine [22], it may be that FAK impairs radiation-induced expression of the genome integrity maintenance genes in SCC cells *via* c-Myc-mediated repression. However, we noted that BRCA1 provides an example of a DNA-damage responsive gene that is only minimally affected by FAK loss; indeed, BRCA1 expression is suppressed by FAK deficiency at later times after irradiation (**Fig. 7D**). Thus, we conclude that radiation-induced transcription of a sub-set of p53-responsive genes is modulated by the presence or absence of FAK, and so is not simply due to general p53 dysfunction.

**Figure 8:**
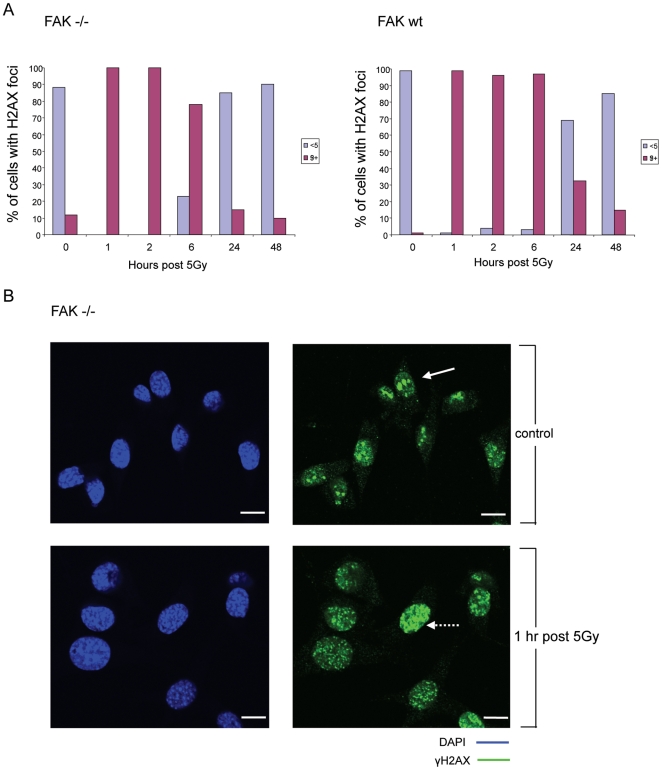
FAK −/− cells are more efficient at resolving DNA damage. (**A**) FAK −/− and FAK wt cells were plated at low density on glass coverslips, incubated for 24 hours and irradiated with 5 Gy. At various time points, the cells were fixed, permeabilised, stained with anti-phospho-γH2AX (serine 139) and visualised using confocal microscopy. The number of foci per nucleus (<5 foci or ≥5 foci) was documented in at least 100 cells. Results shown are representative of one of two separate experiments. (**B**) Representative images demonstrate un-irradiated and irradiated FAK −/− cells at 1 hour post 5 Gy are shown, green – phospho-γH2AX and blue – DAPI (scale bar, 20 µm), arrow in top right hand box shows at a nucleus with <5 foci and broken arrow in bottom right hand box pointing at a nucleus with ≥5 foci.

As mentioned, there was no readily detectable apoptosis or differential cell cycle arrest that can be attributed to FAK status. Hence, we next examined phosphorylation on serine 139 of Histone γH2AX that occurs in response to ionizing radiation [48] and is considered to be a reliable surrogate of double strand break repair. Quantification of the percentage of nuclei containing <5 or ≥5 γH2AX foci showed that both FAK −/− and FAK wt populations had ≥5 γH2AX foci in virtually all cells 1 hour post irradiation with 5 Gy (**Fig. 8A**). However, the FAK −/− cells started to clear these foci within 6 hours and these returned to baseline level within 24 hours; in contrast FAK re-expression in the FAK wt cells caused a slower foci clearance rate (compare 6 and 24 hour time points, (**Fig. 8A**). This is consistent with more efficient DNA repair activity in the FAK −/− cells, and a slower rate of repair when FAK is present. Representative images of γH2AX immunofluorescence in FAK −/− cells (before and 1 hour after 5 Gy irradiation) are shown (**Fig. 8B**). We also found that FAK −/− SCC cells appeared to have generally higher levels of γH2AX foci under control conditions (0 hours) than their FAK-expressing counterparts (images not shown), with most FAK −/− cells displaying several foci and around 10–15% displaying ≥5 foci (**Fig. 8A**, 0 hours). This suggests that FAK-deficient SCC cells may have greater genetic instability than their FAK wt counterparts; at least it appears that the FAK −/− SCC cells have generally enhanced DNA repair functions and this correlates with radio-resistance. Interestingly, we did not find any difference in FAK-dependent regulation of radiation-induced phosphorylation of either p53 or Chk2 (**Fig. S5**).

## Discussion

We here show that, in stark contrast to a previous report in which FAK knock-down sensitised pancreatic cancer cells to ionizing radiation [43], FAK deletion (and a FAK kinase inhibitor) can suppress signalling to radiation-induced, p53-mediated induction of p21, and this is linked to radio-resistance in advanced SCC cells. We think it is important to record that FAK's role in cellular responses to ionizing radiation, and perhaps pro-survival signalling in general, may be context dependent, and that there needs to be caution when considering therapeutic combinations of FAK inhibitors and radiotherapy, as this may not always be clinically beneficial.

In the work described here, we show that deleting FAK (or inhibiting its kinase activity) can release constraints FAK places on signalling from p53 to the induction of several target genes, namely p21 and at least a sub-set of p53-regulated genes involved in DNA repair in SCC cells. Moreover, FAK −/− SCC cells appear to be more efficient at repair after radiation-induced DNA damage. However, in these cells we did not find any significant effect of FAK deletion on p53 protein stability (whether in response to irradiation or DNA damaging drugs), p53 phosphorylation or the ability of p53 to translocate to the nucleus, and we could not find evidence of a FAK/p53 complex that has been reported to operate in other contexts. Thus, our work adds to a growing body of evidence that there is functional cross-talk between the FAK and p53 signalling pathways, but demonstrates that there are additional ways in which this can occur. Although we do not fully understand the mechanism, we found that in the SCC cells from which we could delete FAK by genetic recombination, FAK functions to suppress the radiation-induced DNA repair functions of p53 by blocking induction of p21, and that this is linked to enhanced resistance to ionizing radiation upon loss of FAK signalling.

## Materials and Methods

### Cell culture

Squamous carcinoma cells (SCC) were isolated from chemically induced skin tumours removed from K14*Cre*ER FAK^flox/flox^ transgenic FVB mice. Skin carcinomas were induced using a two-stage chemical carcinogenesis protocol as previously described [25]. Cells were grown in growth media (DMEM, 10% foetal bovine serum and 2 mM glutamine) and maintained in a dry 5% CO_2_ incubator at 37°C, and sub-cultured using standard trypsinisation procedures.

### Subcutaneous tumour growth

Cells were trypsinised, washed in Hanks Balanced Saline Solution (HBSS; Invitrogen, Paisley, UK), and re-suspended at a concentration of between 2.5×10^5^ and 1×10^6^ cells/100 µl in HBSS. 100 µl of cell suspension was injected into the flanks of immune-compromised CD1 nude mice (Charles River Ltd, Margate, Kent, UK) and tumour growth measured every two to three days using callipers. Tumour volume measurements were taken from at least 8 mice for each cell line.

### Irradiation of cells and mice

Cells were grown to 70% confluency and exposed to γ-irradiation from a cobalt (_60_Co) source (Alcyon II teletherapy unit, General Electric, France). The receptacles were set at a distance of 80 cm from the source and a Perspex layer added to the surface of the receptacles in order to achieve build-up. The average dose rate was 1–1.20 Gy/minute and doses of 1–30 Gy were applied. Mice with tumours were treated with whole body irradiation using the cobalt source described.

### Clonogenic assay (limiting dilution method)

Cells were passaged at 70% confluence, counted, and diluted in complete growth medium to yield a final concentration that would permit single colony growth after the required period of incubation. 100 µl of this cell suspension was added to each well of a flat bottomed 96 well microplate. The plates were incubated for 6 hours to allow cell adhesion then irradiated at increasing doses of irradiation. After 7 days (to allow 6 cell doubling times), the plates were washed in PBS, fixed in methanol, and stained with crystal violet (Sigma Chemical Co, Poole, UK). The numbers of colonies present per plate were counted on low power bright field microscopy. Plating efficiency was determined by dividing the number of colonies present by the total number of cells seeded per plate. Surviving fraction was then calculated by dividing the plating efficiency at each radiation dose by the plating efficiency of unirradiated cells. Each experiment was performed in triplicate and on at least 3 separate occasions. The data was combined and displayed graphically as mean ± SEM.

### Transient transfection with siRNA

The Dharmacon Smartpool method (Dharmacon, Abgene Ltd., Epsom, UK) of mammalian cell transfection was used for the transfection of sub-confluent SCC 7.1 FAK −/− and FAK wt cells. The siRNA for transfection was diluted in sterile PBS and added to serum free MEM containing 5 µl of Dharmafect Transfection Reagent 1 to give a total volume of 400 µl. The complexes were incubated at room temperature for 20 minutes then added directly to wells containing 1600 µl of complete medium (final concentration of siRNA, 100 nM). The plates were incubated for 24 hours at 37°C in an atmosphere of 95% air and 5% CO_2_ prior to any cell treatments and harvesting. In each experiment, a scrambled pool of siRNA was used as control.

### Immunohistochemsitry and immunofluorescence

Fixed paraffin embedded tissue sections mounted on slides were dewaxed in xylene solution followed by stepped rehydration via a series of graded alcohols to water. Antigen retrieval was then performed by boiling the slides in sodium citrate solution (pH 6) for 20 minutes. The slides were incubated with peroxidase block for 5 minutes to quench endogenous peroxidase activity, blocked in 10% FBS in 0.01 M Tris buffered saline (pH 7.5) for one hour at room temperature, and incubated with p21 antibody (SC-421 (Autogen bioclear (Wiltshire UK) at 1∶800 dilution) overnight at 4°C. A non-immune IgG control was compared in parallel to the investigated sections by omitting the primary antibody step. Visualisation was carried out with a DAKO EnVision kit ™ (Dako UK Ltd, Ely, UK) as per manufacturers' instructions. The resulting sections were analysed and images captured digitally using an Olympus BX51 microscope and cell∧D software (Olympus UK Ltd, Hertfordshire, UK). For phospho-γH2AX staining, cells were fixed in 4% paraformaldehyde for 15 minutes. Permeabilisation was with 0.2% Triton X-100 in PBS for 5 minutes, staining with primary antibody (anti-phospho-gH2AX (1∶250; Upstate (Millipore), Hampshire, UK)) and treatment with secondary antibody conjugated to Alexa® 488 or 594 fluorescent dyes (Invitrogen, Paisley, UK) at a 1/200 dilution for one hour. Cells were visualised by confocal microscopy.

### Protein analysis: SDS-PAGE and Western blotting

Confluent cells were harvested in RIPA (50 mM Tris/HCl, pH 7.4, 150 mM NaCl, 1% Sodium Deoxycholate, 1% NP40, 5 mM EGTA plus standard protease inhibitor cocktail) lysis buffer. Cell lysates were centrifuged in a bench-top, refrigerated centrifuge at 13000 rpm at 4°C and the supernatant retained. The cell lysates were then snap frozen on dry ice and stored at −80°C. Animal tissue was removed post mortem and immediately frozen in liquid nitrogen for storage at −80°C. At the required time the frozen tissue was added to a Precellys tube (Bertin Technologies, Provence, France) with 100 µl of ice-cold T-PER buffer. The samples were homogenised (Precellys 24 device – Bertin Technologies, Provence, France), transferred to a 1.5 ml Eppendorf® tube and centrifuged as outlined above. Protein concentration was determined using the MicroBCA™ Protein Assay Kit (PERBIO, Glasgow, UK) and quantified by measuring light absorbance with a DU® 650 spectrophotometer at a wavelength of 562 nm (Beckman Coulter UK Ltd, Buckinghamshire, UK). Proteins were separated on SDS-polyacrylamide gels; specifically, protein lysates of 5–40 µg were denatured and reduced by addition of NuPAGE® 4× LDS sample buffer (Invitrogen, Paisley, UK). The samples were boiled for 5 min and then loaded directly to an appropriate well of a NuPAGE® Bis-Tris polyacrylamide gel immersed in Invitrogen™ NuPAGE® MOPS SDS running buffer. 10%, 12% or 4–12% gradient gels were used depending on the molecular weight of the protein of interest. The gels were run at 200 V for 1 h. For western blotting, separated proteins were transferred to a nitrocellulose membrane using wet blotting apparatus (Jencons, Leighton Buzzard, UK) with an applied voltage of 30 V for 90 minutes, blocked in 5% bovine serum albumin (BSA), re-constituted in 20 mM Tris-Cl; pH 7.6, 150 mM NaCl and 0.1% Tween20 (TBST), for one hour at room temperature with gentle agitation. The primary antibody (FAK (New England Biolabs) – 1∶1000; β-actin (Sigma) – 1∶5000; p21 – 1∶1000; p53 (C12 New England Biolabs) – 1∶1000); phospho-Chk2 (S68 New England Biolabs) – 1∶1000); phospho-p53(IC12 New England Biolabs) – 1∶500 was added at the dilutions stated overnight at 4°C. The membrane was then washed several times with TBST before the application of the appropriate horseradish peroxidase (HRP) conjugated anti-immunoglobulin G (IgG) secondary antibody diluted 1∶5000 in 5% BSA - TBST solution. Detection was by Amersham Biosciences (Little Chalfont, UK) enhanced chemiluminescence (ECL).

### Extraction of RNA and qRT-PCR analysis

RNA was isolated from 1×10^6^ cells using miRNeasy mini RNA extraction kit (Qiagen, Crawley, UK). 1 µg of RNA was then converted to cDNA using Superscript First-Strand cDNA synthesis kit (Qiagen, Crawley, UK). The cDNA was diluted 1 in 5 then prepared for qRT-PCR analysis by adding 5 µl to 45 µl SybrGreen master mix (Invitrogen, Paisley, UK) containing 1 µM of paired validated primers directed against the target gene of choice (Qiagen, Crawley, UK). All primer pairs were assessed for linearity prior to use and produced a PCR single product of the correct size as outlined by the manufacturer. Real time PCR was performed on a gradient cycler (Bio-Rad, Hertfordshire, UK) with the following programme: 95°C for 15 minutes (1 cycle); 95°C for 15 seconds+55°C for 30 seconds+72°C for 30 seconds (39 cycles); 72°C for 5 minutes (1 cycle); melting curve 70–95°C, hold every 0.1 seconds; 72°C for 10 minutes (1 cycle); 15°C for 10 minutes (1 cycle). Data was analysed using Opticon software V3.1 (Bio-Rad, Hertfordshire, UK). Beta-actin controls were included with each reaction to act as a housekeeping gene and fold change in mRNA levels calculated by the ddC(t) method {Livak, 2001 #476}. The samples were loaded in triplicate and the mean ± SEM from three combined experiments displayed graphically. Primers for p21 were **5′-3′** AGC CTG ACA GAT TTC CAC and **5′-3′** CTT TAA GTT TGG AGA CTG GGA (provided by VH Bio, Gateshead, UK); primers for ddb2, gadd45, p53R2, Brca1, PUMA and β-actin genes are Quiagen Quantifect Validated Primer Pairs for which the sequences were not disclosed to us.

### Statistical analysis

Graphs and bar charts were created in Excel and represent the mean value ± SD or mean value ± SEM from three separate experiments. Statistical tests were performed in Minitab 15, p<0.05 was considered significant and is denoted by *. An unpaired t-test was used to compare the means of two populations with approximately equal variance and normal distribution, where n = number of data sets that contributed towards the mean. For the purposes of IHC data analysis, the mean percentage of positively stained cells per xenograft was calculated based on examination of twenty high powered fields. The mean values of two test groups were analysed by either the Kruskal-Wallis test or the Mann Whitney U test (typically three to five xenografts from separate mice were included in each defined experimental group).

## Supporting Information

Figure S1 **p21, but not p53, induction after irradiation of SCC cells is dependent on FAK status.** Densitometric quantification of p21 at times after 5 Gy irradiation of SCC cells (**A**; upper panel) and of p53 at 2 hours after various radiation doses or following overnight treatment with 0.1 M actinomycin D (**B**; lower panel).
(TIF)

Figure S2 **There is no interaction between FAK and p53 in SCC cancer cells.** (**A**) FAK wt cells were irradiated at around 70% confluency and lysates prepared at 0 (FAK wt) and 2 hours (FAK wt+5 Gy). 1 mg of protein was immunoprecipated with an anti-FAK agarose conjugated antibody at 4°C overnight. The IPs were separated by SDS-PAGE and immunoblots probed with anti-FAK (upper panel), anti-Src (middle panel), and anti-p53 (lower panel). As a negative control, irradiated FAK wt cell lysates were also immunoprecipitated with an anti-histidine agarose conjugated antibody. (**B**) 20 µg of protein lysates were immunoblotted and probed with anti-FAK, anti-Src, anti-p53, and anti-β-actin.
(TIF)

Figure S3 **FAK loss does not promote radioresistance by increasing the length of cell cycle arrest in response to ionising radiation.** FAK −/− (**A**) and FAK wt (**B**) cells were irradiated with 5 Gy at 70% confluence; at various time points samples were fixed in 70% ethanol, stained with propidium iodide and subjected to cell cycle analysis. The percentage of gated cells in each of the component phases (G1, S, and G2/M) of the cell cycle was evaluated at each time point. The graphs shown represent the mean ± SEM from three experiments.
(TIF)

Figure S4 **PUMA is stimulated in FAK −/− cells after irradiation.** (**A**) RNA was extracted from subconfluent FAK −/− and FAK wt cell populations at various time points after 5 Gy irradiation. qRT-PCR analysis was then performed as previously described using PUMA primers with β-actin as a loading control. (**B**) FAK −/− and FAK wt cells were irradiated with 5 Gy at 70% confluence and lysates prepared at the indicated time points. Immunoblotting was then performed with anti-PUMA (upper) and anti-β-actin (lower) antibodies. Species corresponding to PUMA-α and PUMA-β are shown.
(TIF)

Figure S5 **Phosphorylation of p53 and Chk2 after irraditaion are similar in FAK-proficient and FAK-deficient SCC cells.** Subconfluent populations of FAK −/− and FAK wt cells were irradiated with 5 Gy and protein extracts were prepared at various time points. The extracts were then separated by SDS-PAGE, transferred to nitrocellulose, and probed with anti-phosph-p53, anti-p53, and anti-β-actin as indicated (**A**), and anti-phospho-Chk2 and anti-β-actin (**B**).
(TIF)

Methods S1 **Supplementary methods are provided for immunoprecipitation and cell cycle analysis.**
(DOCX)
