# Supplementary figures and images for: FAK Deletion Promotes p53-Mediated Induction of p21, DNA-Damage Responses and Radio-Resistance in Advanced Squamous Cancer Cells
Source: PLoS One. 2011 Dec 14;6(12):e27806. doi: 10.1371/journal.pone.0027806 (PMC3237418; doi:10.1371/journal.pone.0027806)

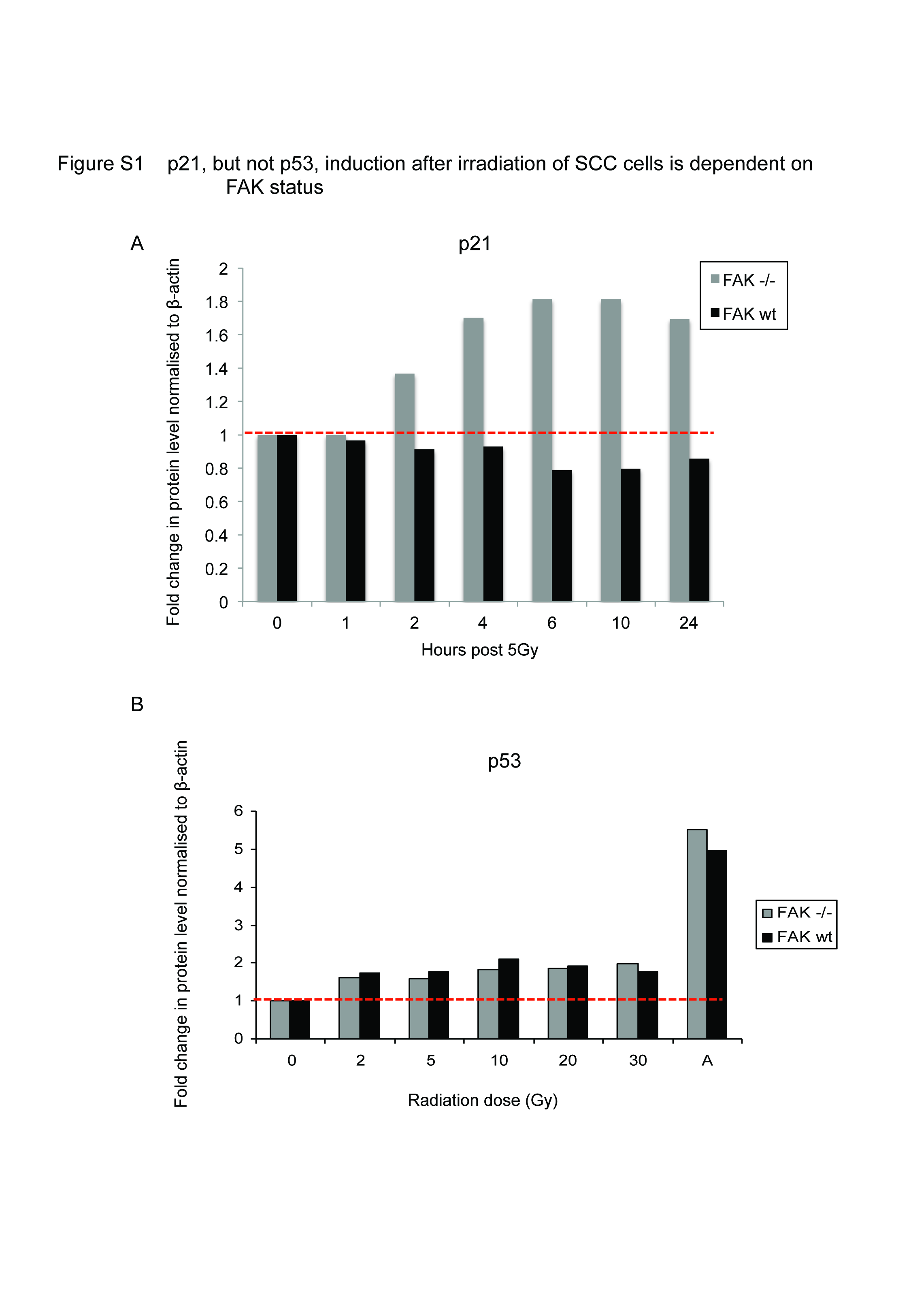

Supplement: Figure S1 — p21, but not p53, induction after irradiation of SCC cells is dependent on FAK status. Densitometric quantification of p21 at times after 5 Gy irradiation of SCC cells (A; upper panel) and of p53 at 2 hours after various radiation doses or following overnight treatment with 0.1 M actinomycin D (B; lower panel). (TIF) [file pone.0027806.s001.tif]

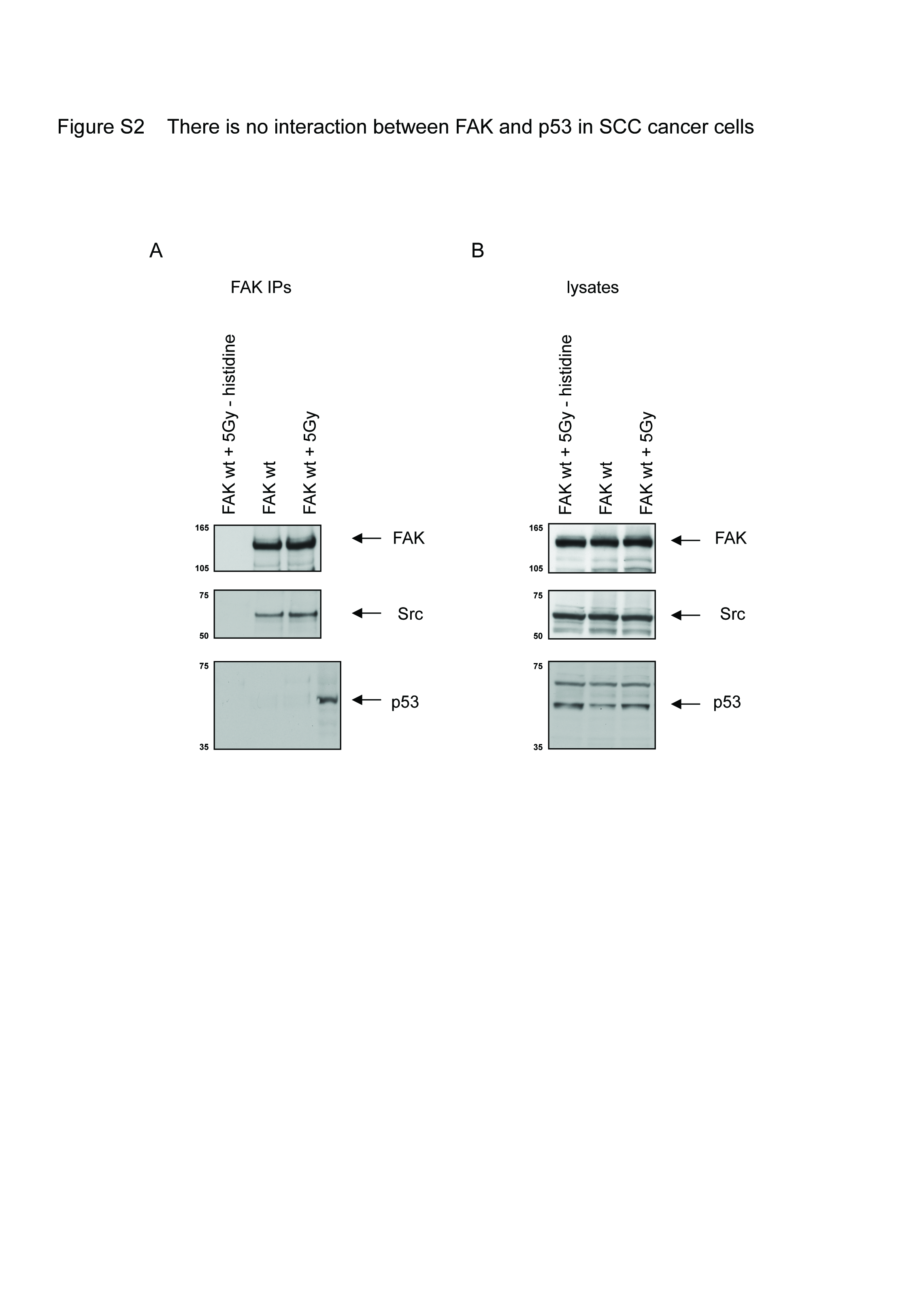

Supplement: Figure S2 — There is no interaction between FAK and p53 in SCC cancer cells. (A) FAK wt cells were irradiated at around 70% confluency and lysates prepared at 0 (FAK wt) and 2 hours (FAK wt+5 Gy). 1 mg of protein was immunoprecipated with an anti-FAK agarose conjugated antibody at 4°C overnight. The IPs were separated by SDS-PAGE and immunoblots probed with anti-FAK (upper panel), anti-Src (middle panel), and anti-p53 (lower panel). As a negative control, irradiated FAK wt cell lysates were also immunoprecipitated with an anti-histidine agarose conjugated antibody. (B) 20 µg of protein lysates were immunoblotted and probed with anti-FAK, anti-Src, anti-p53, and anti-β-actin. (TIF) [file pone.0027806.s002.tif]

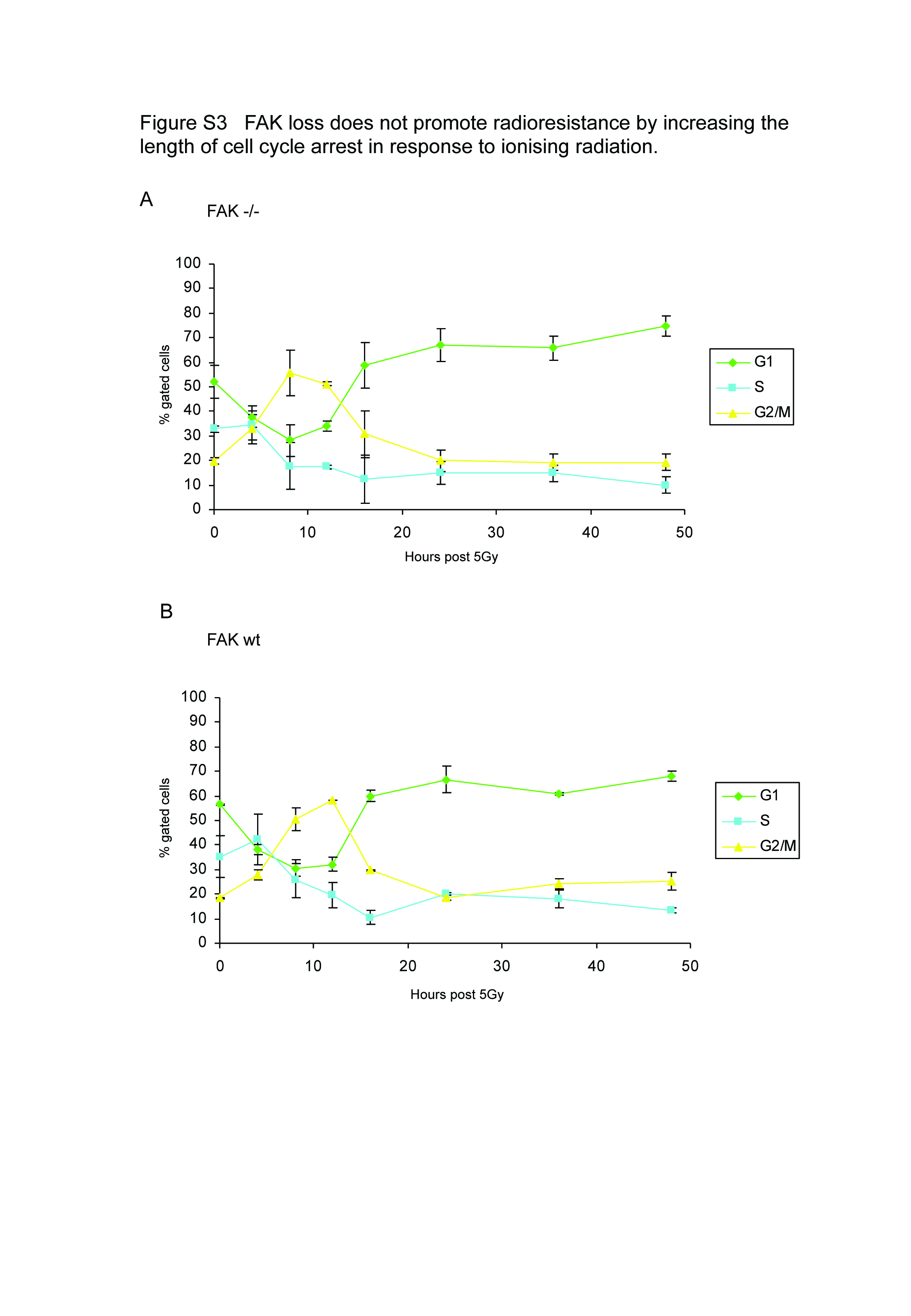

Supplement: Figure S3 — FAK loss does not promote radioresistance by increasing the length of cell cycle arrest in response to ionising radiation. FAK −/− (A) and FAK wt (B) cells were irradiated with 5 Gy at 70% confluence; at various time points samples were fixed in 70% ethanol, stained with propidium iodide and subjected to cell cycle analysis. The percentage of gated cells in each of the component phases (G1, S, and G2/M) of the cell cycle was evaluated at each time point. The graphs shown represent the mean ± SEM from three experiments. (TIF) [file pone.0027806.s003.tif]

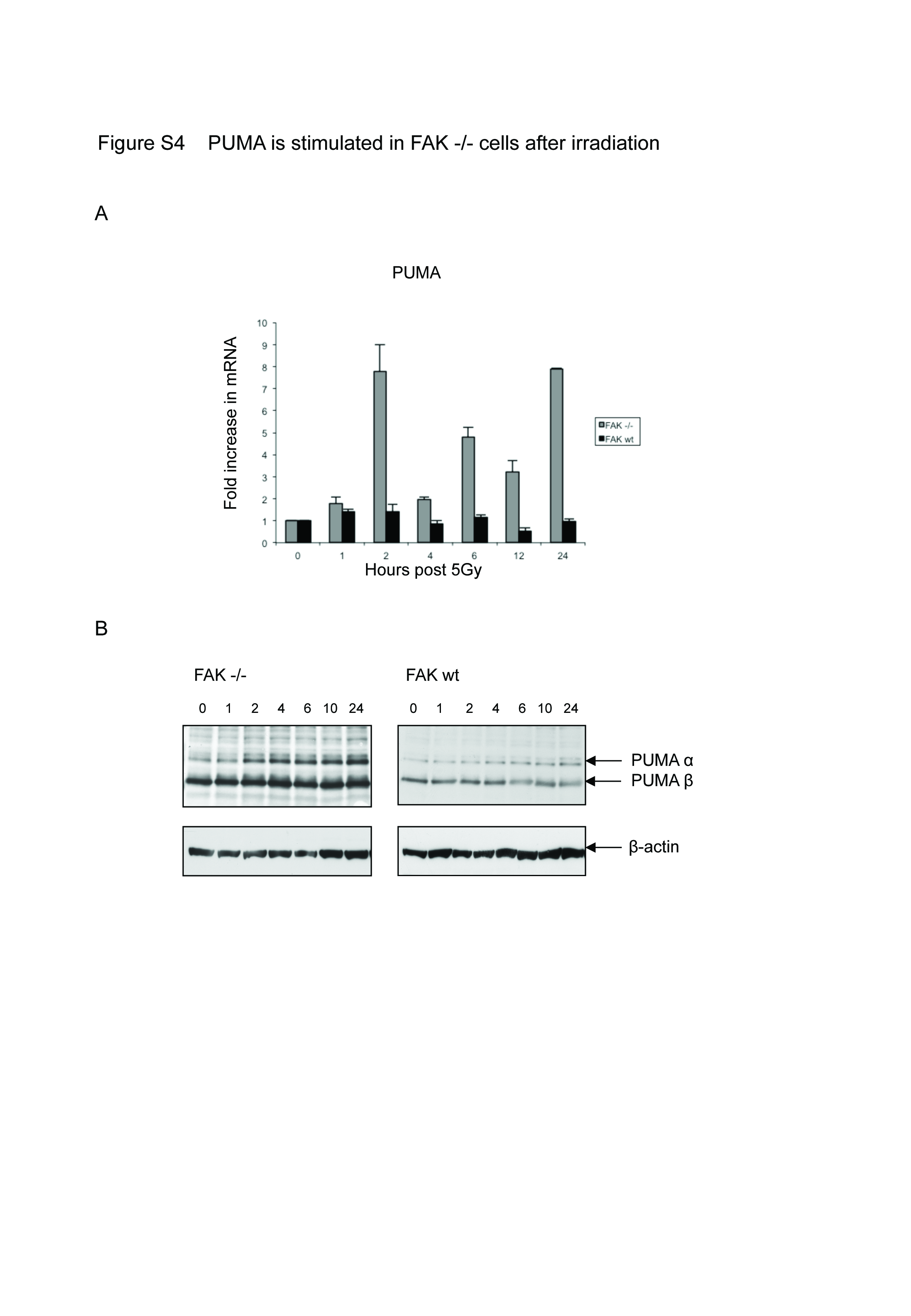

Supplement: Figure S4 — PUMA is stimulated in FAK −/− cells after irradiation. (A) RNA was extracted from subconfluent FAK −/− and FAK wt cell populations at various time points after 5 Gy irradiation. qRT-PCR analysis was then performed as previously described using PUMA primers with β-actin as a loading control. (B) FAK −/− and FAK wt cells were irradiated with 5 Gy at 70% confluence and lysates prepared at the indicated time points. Immunoblotting was then performed with anti-PUMA (upper) and anti-β-actin (lower) antibodies. Species corresponding to PUMA-α and PUMA-β are shown. (TIF) [file pone.0027806.s004.tif]

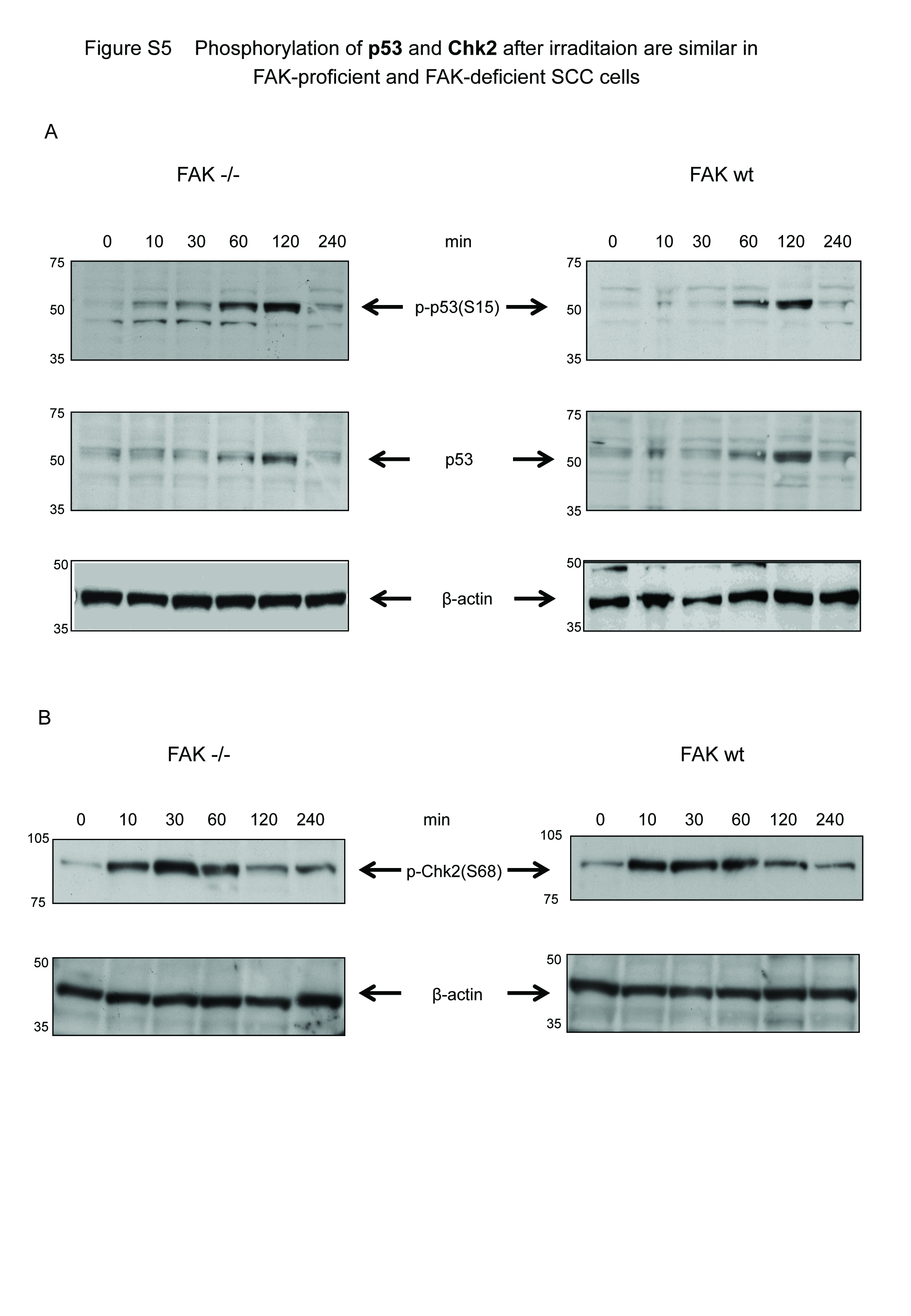

Supplement: Figure S5 — Phosphorylation of p53 and Chk2 after irraditaion are similar in FAK-proficient and FAK-deficient SCC cells. Subconfluent populations of FAK −/− and FAK wt cells were irradiated with 5 Gy and protein extracts were prepared at various time points. The extracts were then separated by SDS-PAGE, transferred to nitrocellulose, and probed with anti-phosph-p53, anti-p53, and anti-β-actin as indicated (A), and anti-phospho-Chk2 and anti-β-actin (B). (TIF) [file pone.0027806.s005.tif]
