# Supplementary material for: FAK Deletion Promotes p53-Mediated Induction of p21, DNA-Damage Responses and Radio-Resistance in Advanced Squamous Cancer Cells
Source: PLoS One. 2011 Dec 14;6(12):e27806. doi: 10.1371/journal.pone.0027806 (PMC3237418; doi:10.1371/journal.pone.0027806)
Supplement: Methods S1 — Supplementary methods are provided for immunoprecipitation and cell cycle analysis. (DOCX) [file pone.0027806.s006.docx]

**Methods S1**

***Immunoprecipitation***

500µg of whole cell lysate was pre-cleared by adding 20µl protein-G sepharose slurry (Sigma Chemical Co, Poole, UK) and rotating at 4^o^C for one hour. Following this, the lysate was transferred to a clean tube and incubated with 10µl anti-FAK agarose conjugated primary antibody (Chemicon International, Harrow, UK) overnight at 4^o^C. As a negative control, lysate was immunoprecipitated with an anti-histidine agarose conjugated antibody (Sigma Chemical Co, Poole, UK). The antibody-protein complexes were then washed in RIPA buffer and resuspended in sample buffer prior to western blot analysis.

***Cell cycle analysis***

Cells were treated as indicated, trypsinised, and washed twice in ice cold PBS prior to fixation in 70% ethanol/PBS overnight at 4^o^C. For DNA content analysis (including sub-2n DNA), cells were pelleted and resuspended in PBS containing 1µg/ml RNase A (Qiagen, Crawley, UK) and 10µg/ml propidium iodode (PI) (Sigma Chemical Co, Poole, UK), incubated at room temperature for 30 minutes, then passed through a FACSCalibur flow cytometer with CELLQuest software (Becton Dickinson, Oxford, UK). The data was analysed on FloJo (Treestar, Stanford, CA, USA) and a graphical representation obtained. Experiments were performed at least 3 times and the data presented represents the combined mean ± SEM, unless stated otherwise.
